# The development of search filters for adverse effects of surgical interventions in medline and Embase

**DOI:** 10.1111/hir.12213

**Published:** 2018-03-31

**Authors:** Su Golder, Kath Wright, Yoon Kong Loke

**Affiliations:** ^1^ Department of Health Sciences University of York York UK; ^2^ CRD University of York York UK; ^3^ School of Medicine University of East Anglia Norwich UK

**Keywords:** EMBASE, information retrieval, literature searching, medical subject headings (MeSH), medline, meta analysis, methodological filters, review, systematic, search strategies, searching

## Abstract

**Background:**

Search filter development for adverse effects has tended to focus on retrieving studies of drug interventions. However, a different approach is required for surgical interventions.

**Objective:**

To develop and validate search filters for medline and Embase for the adverse effects of surgical interventions.

**Methods:**

Systematic reviews of surgical interventions where the primary focus was to evaluate adverse effect(s) were sought. The included studies within these reviews were divided randomly into a development set, evaluation set and validation set. Using word frequency analysis we constructed a sensitivity maximising search strategy and this was tested in the evaluation and validation set.

**Results:**

Three hundred and fifty eight papers were included from 19 surgical intervention reviews. Three hundred and fifty two papers were available on medline and 348 were available on Embase. Generic adverse effects search strategies in medline and Embase could achieve approximately 90% relative recall. Recall could be further improved with the addition of specific adverse effects terms to the search strategies.

**Conclusion:**

We have derived and validated a novel search filter that has reasonable performance for identifying adverse effects of surgical interventions in medline and Embase. However, we appreciate the limitations of our methods, and recommend further research on larger sample sizes and prospective systematic reviews.

**Key Messages**

- Searches with generic adverse effects terms as suggested in this paper achieve approximately 90% relative recall in either medline or Embase.
- The addition of specific named adverse effects search terms in either medline or Embase is likely to further improve relative recall.
- Any search with adverse effects terms is unlikely to achieve 100% recall as a few articles still do not indicate in the title, abstract or indexing that the full paper contains adverse effects data.
- The relative recall achieved from searching with adverse effects terms for surgical interventions is similar to that for drug interventions.

## Introduction

Any intervention that has an effect can have an adverse effect. Searching for information on adverse effects can be problematic. This is because there are a number of specific challenges which need to be overcome. The first problem is that adverse effects are generally considered to be either a secondary or even tertiary outcome in health research (Golder & Loke, 2012a,b,c). Consequently, the reporting of adverse effects in the title and abstract of database records corresponding to relevant papers has been poor and in turn this has also led to the poor indexing of such records. However, research in the arena of drug interventions suggests that the situation is improving (Golder & Loke, 2012a,b,c). Secondly, the terminology surrounding adverse effects has also been notoriously inconsistent and this again is reflected in the indexing of database records. Thirdly, another issue is that not all adverse effects are known at the time of searching. The aim of the search, for example, might be to develop a safety profile for an intervention with identification of the adverse effects as one of its primary objectives. In such instances reliance on generic adverse effects terms (such as ‘side‐effects’, ‘complications’, ‘harm’) becomes more imperative. Where information on specific adverse effects is sought, terms for the specific adverse effects (such as ‘pain’, ‘fatigue’ or ‘mortality’) may be used either instead of or in addition to generic search terms. Lastly, it is common to include all study designs for adverse effects or at least include study designs beyond randomised controlled trials (RCTs). Whilst search filters for RCTs have made it easier to search for RCTs, searching beyond RCTs is still problematic.

One way in which searching for adverse effects could be made easier is through the development of search filters. Search filters are combinations of search terms which are designed to improve the efficiency and effectiveness of searching. The majority of search filters are designed to identify a particular topic (such as a condition or population) or study design (such as RCTs). Search filter development in the arena of adverse effects has tended to focus on identifying studies that report on adverse drug effects (Badgett, Chiquette, Anagnostelis & Mulrow, 1999; Golder & Loke, 2012a,b,c; Golder, McIntosh, Duffy & Glanville, 2006; Wieland & Dickersin, 2005). However, a different approach is required for non‐drug adverse effects (Farrah, Mierzwinski‐Urban & Cimon, 2016; Golder, Wright & Rodgers, 2014). Recent research has indicated that, whilst a filter for all types of non‐drug interventions may not be feasible, a filter specifically aimed at identifying adverse effects of surgical interventions is feasible (Golder, Wright & Loke, 2017).

Whilst the adverse effects of drug interventions are the most commonly researched in systematic reviews (237/348, 68%), the adverse effects of surgical interventions are the next most popular type of intervention evaluated (80/348, 23%) (Golder, Loke, Wright & Sterrantino, 2016; Golder, Loke & Zorzela, 2013, 2014). Surgical interventions that are evaluated in systematic reviews include procedures that are very widely used such as caesarean section and breast reconstruction. At present no search filters for the adverse effects of surgical interventions are available.

Search filters may be useful not only for librarians and information professionals. There are clinicians involved in surgical practice who are not expert searchers but would like a relatively efficient method of retrieving clinically useful information for their practice. Equally, policy makers as well as those who write guidelines need a simple way of accessing relevant material so that they can generate appropriate advice on the benefit:harm of surgical interventions and retrieve data that helps keep them fully informed of any risks.

We therefore aimed to objectively identify high performing search terms and approaches for identifying relevant studies for surgical adverse effects with the ultimate aim of creating validated search filters for the adverse effects of surgical interventions in medline and Embase.

## Methods

### Systematic review identification

Systematic reviews of adverse effects were identified by manually screening all records published in 2014 in the Database of Abstracts of Reviews of Effects (DARE) (via the Centre for Reviews and Dissemination website, April 2015). No automated search strategy was implemented, as previous research has indicated that even very broad search strings would miss relevant records (Golder, McIntosh & Loke, 2006). The DARE database was chosen because it was the most accessible major collection of systematic reviews of health care interventions. DARE was compiled through rigorous monthly searches of bibliographic databases, including medline and Embase, as well as handsearching of key journals, grey literature, and regular searches of the Internet. It also contains all Cochrane reviews, both new and updated. DARE ceased production in March 2015 but continues to be available in archive format. A systematic review was considered eligible for inclusion if:

- Adverse effect(s) for a surgical intervention were the primary outcome.
- No adverse effects search terms (either generic, such as ‘adverse effects’ or ‘side effects’ or named, such as ‘fatigue’ or ‘insomnia’) had been used by the review authors. Typically, such reviews rely on search terms for the population or condition and intervention only. This enabled an unselected cohort to be built, where relevant articles are not chosen because of the presence of adverse effects terms.
- The search included either handsearching or reference checking in addition to database searches.

The author and another researcher independently screened titles and abstracts in DARE and selected systematic reviews for potential inclusion. Any discrepancies between the researchers were resolved by discussion and consensus. The full articles of potentially relevant systematic reviews were also independently screened, with again discrepancies resolved by discussion and consensus.

### Included primary studies

The full text of the included articles within these systematic reviews was checked to confirm the presence of adverse effects data that had been used in the systematic review. The use of included papers from systematic reviews has been shown to be effective in identifying a reference standard set of records for the use in studies evaluating search strategies (Sampson et al., 2006).

The first stage of the analysis was to check whether each paper was contained in medline and/or Embase by using several search iterations as necessary of the author names and/or words from the title. Each set of records available on each database were then divided randomly into three sets – one development set, one evaluation set and one validation set using random numbers generated by RANDOM.ORG.

In each database, the development set of records was used to identify search terms for the development of the search filter and the evaluation set of records was used to test the search filter developed. The validation set was then used to test the retrieval performance of the search filter. Sampson et al. (2006) state that a gold standard of 100 publications is sufficient for the development of search filters. We therefore aimed to identify a minimum of 100 records for the development set in both medline and Embase.

Individual word and multiple‐word frequency analysis on the development set was undertaken using WriteWords to identify commonly occurring terms related to adverse effects. WriteWords is freely available on the Internet and allows you to count the frequency of usage of words or phrases in text (http://www.writewords.org.uk/phrase_count.asp). We calculated relative recall as a measure of the percentage of known records retrieved using the filter because it provides an estimate of sensitivity. The relative recall of the relevant search terms identified from frequency analysis was calculated using the following formula;

### Relative recall calculation

$$\frac{\text{No of relevant records retrieved}}{\text{No of relevant records available}}\times 100=\text{Relative recall as a percentage}\;\left(\%\right)$$

This is slightly different to sensitivity which is a measure of the number of relevant records retrieved over the total number of relevant records (Sampson et al., 2006). However, these terms are often used interchangeably in the literature (Sampson et al., 2006).

In order to give a relative or rank estimate of the precision of the search terms, we also identified the total number of records that would be retrieved from medline or Embase at the time of conducting the present research using the search term. We then calculated an approximation of the *relative* precision of the term in relation to the other terms we identified. This calculation was used merely to give an approximate comparison of precision between the relevant search terms.

A draft filter was created after testing combinations of relevant search terms and search phrases identified in the development set. We started by using the search term with the highest recall and then tested all other terms with a frequency above two to ascertain the incremental increase in recall when added to the first term. This process continued until no more new records were being identified by additional search terms. We tested this process by using several other iterations of combinations of search terms.

The filter with the highest relative recall when applied to the records in the development set was next applied to the evaluation set and then after any necessary modifications was applied to the validation set.

This process was first undertaken in medline and then repeated in Embase. We also examined those records not retrieved by our generic search terms and ascertained whether specific adverse effects search terms (such as ‘fatigue’ or ‘insomnia’ etc.) would have been successful in retrieval. We also noted any database records with no indication that the full text contained information on adverse effects.

## Results

From 9129 DARE records screened, 451 full reports were retrieved and of these 348 reviews were about adverse effects with 79 evaluating surgical interventions. Of these 79 reviews, only 19 did not include any adverse effects terms in their search strategies and had carried out reference checking and/or hand searching. These 19 reviews included 358 unique studies. Of these 358 studies, 352 were available on medline and 348 were available from Embase.

### medline

The 352 records comprising the gold standard set on surgical adverse effects available in medline were randomly allocated into a development set with 118 records and an evaluation and validation set of records containing 117 records each.

#### Development of the medline search strategy

The development set was used to identify the search terms or phrases for the filter. Word and phrase frequency analysis on the development set was undertaken using WriteWords. We started by searching the search term with the highest recall, ‘complication*’ in the title and abstract (Table 1). We then tested all other potentially relevant terms with a frequency above two to ascertain the incremental increase in recall when added to the first term. This process continued until no more new records were being identified by additional search terms.

**Table 1:** Search terms in medline development set of records (in order of relative recall)

Search term	Fields searched	Relative recall (*n* = 118)	Total number of records retrieved medline 1946 to Present (11/04/2017)	Approximate relative precision estimate (%)
Complication^a^	Title/abstract	64% (75)	667 876	0.011
Complications	Title/abstract	53% (62)	525 095	0.012
Safe^a^	Title/abstract	40% (47)	593 190	0.008
Adverse effects (ae)	Subheading	36% (43)	1 560 655	0.003
Post‐operative complications	MeSH	26% (31)	323 145	0.010
Safe	Title/abstract	26% (31)	251 678	0.012
Complication	Title/abstract	24% (28)	205 489	0.014
Complications (co)	Subheading	20% (24)	1 812 415	0.001
Safety	Title/abstract	17% (20)	334 856	0.006
Procedure‐related	Title/abstract	5% (6)	6447	0.093
Adverse events	Title/abstract	3% (4)	90 365	0.004
Safely	Title/abstract	3% (4)	48 708	0.008
Tolerated	Title/abstract	3% (3)	113 797	0.003
Intraoperative complications	MeSH	2% (2)	29 331	0.007
Risks	Title/abstract	2% (2)	168 332	0.001
Sequelae	Title/abstract	2% (2)	48 870	0.004

a is the truncation symbol in OVID

A search strategy which retrieved 101/118 records (86%) was achieved using the combinations of terms presented in Box 1. Of the 17 records not retrieved by the search strategy 10 had terms related to specific adverse effects (such as ‘wound infection’, ‘mortality’, ‘donor site morbidity’, ‘postoperative pain’ and ‘nausea and vomiting’) while seven gave no indication that the full paper contained information on adverse effects. A search strategy which incorporates both generic and specific adverse effects terms could therefore potentially achieve 94% (111/118) recall.

Box 1 **Search strategy from medline development set**

1 complication*.ti,ab. (75)

2 ae.fs. (89) [adverse effects]

3 safe*.ti,ab. (97)

4 co.fs. (100) [complications]

5 post‐operative complications/(101)

6 or/1–5

#### Evaluation of the medline search strategy

This search strategy (Box 1) was then tested on the evaluation set of records and retrieved 110/117 (94%) records. On inspection of the records that had not been retrieved from the evaluation set, we did not find any additional potentially relevant generic adverse effects terms. Thus, no additional terms were added to the search strategy. However, there were specific adverse effects in two of the seven records that had not been retrieved by this search strategy (MeSH terms such as ‘pain, postoperative/’ and ‘surgical site infection/’ were used). A search strategy which incorporates both generic and specific adverse effects terms could therefore potentially achieve 96% (112/117) recall in the evaluation set of records.

#### Validation of the medline search strategy

The search strategy (Box 1) performed less well on the validation set of records and retrieved 102/117 (87%) of the records.

We conducted post‐hoc analysis to identify factors that may have affected the recall. When we explored the records that had not been retrieved from the validation set, there was one additional record that could have been retrieved if the phrase ‘procedure related’ was added to the search strategy in the title and abstract field. After adding the phrase ‘procedure related’ to the search strategy we then retrieved 103/117 records (88%) of the records in the validation set with the revised search strategy. Of the 14 records not retrieved six contained terms related to specific adverse effects such as ‘postoperative pain’, ‘adverse oncological consequences’, ‘postoperative death’, ‘postoperative mortality’, ‘wound infections’, ‘surgical wound infection/’, and ‘sensory or motor nerve loss’. A search strategy which incorporates both generic and specific adverse effects terms could therefore potentially achieve 93% (109/117) recall in the validation set of records.

The terms which gave the highest precision in medline were estimated to be ‘procedure related’, ‘complication’ and ‘safe’ in the title and abstract.

### Embase

The 348 records comprising the gold standard set on surgical adverse effects available in Embase were randomly divided into a development set of 116 records and an evaluation and validation set with 116 records in each set.

#### Development of the Embase search strategy

The development set was used to identify the search terms or phrases for the filter. Word and phrase frequency analysis on the development set was undertaken using WriteWords. We started by searching the search term with the highest recall ‘complication*’ in the title and abstract (Table 2). We then tested all other potentially relevant terms with a frequency above two to ascertain the incremental increase in recall when added to the first term. This process continued until no more new records were being identified by additional search terms.

**Table 2:** Search terms in Embase development set of records (in order of relative recall)

Search terms	Fields searched	Relative recall (*n* = 116)	Total number of records retrieved Embase 1974 to 2017 April 04	Approximate relative precision estimate (%)
Complication^a^	Title/abstract	63% (73)	1 042 132	0.007
Complications	Title/abstract	58% (67)	821 891	0.008
Complication (co)	Subheading	54% (63)	1 703 285	0.004
Post‐operative complication/	Emtree	40% (44)	308 857	0.014
Safe^a^	Title/abstract	34% (39)	973 026	0.004
Complication/	Emtree	28% (32)	74 111	0.043
Safe	Title/abstract	22% (26)	413 259	0.006
Complication	Title/abstract	22% (25)	329 900	0.008
Safety/	Emtree	13% (15)	407 529	0.004
Safety	Title/abstract	11% (13)	565 638	0.003
Patient safety/	Emtree	8% (9)	90 154	0.010
Surgical risk/	Emtree	5% (6)	37 098	0.016
Adverse drug reaction (ae)	Subheading	4% (5)	1 205 894	0.000
Safely	Title/abstract	3% (4)	78 470	0.005
Post‐operative morbidity	Title/abstract	3% (3)	12 493	0.024
Peroperative complication/	Emtree	2% (2)	34 788	0.006
Adverse events	Title/abstract	2% (2)	172 555	0.001
Procedure‐related	Title/abstract	2% (2)	11 794	0.017
Risks	Title/abstract	2% (2)	248 977	0.008
Safer	Title/abstract	2% (2)	37 655	0.005
Tolerated	Title/abstract	2% (2)	183 044	0.001

a is the truncation symbol in OVID

A search strategy which retrieved 102/116 records (88%) was achieved using the combination of search terms presented in Box 2. Of the 14 records not retrieved by the search strategy four had terms related to specific adverse effects (such as ‘postoperative pain’, ‘death’, ‘mortality’, ‘morbidity’, ‘sensory or motor nerve loss’, and 10 gave no indication that the full paper contained information on adverse effects. A search strategy which incorporates both generic and specific adverse effects terms could therefore potentially achieve 91% (106/116) recall.

Box 2 **Search strategy from Embase development set**

1 complication*.ti,ab. (73)

2 co.fs. (91) [Complication]

3 safe*.ti,ab. (96)

4 ae.fs. (99) [adverse drug reaction]

5 post‐operative morbidity.ti,ab. (100)

6 surgical risk/(101)

7 complication/(102)

8 or/1–7

#### Evaluation of the Embase search strategy

This search strategy (Box 2) was then tested on the evaluation set of records and retrieved 101/116 (87%) records. There were two additional records in this evaluation set that could have been retrieved if the Emtree term ‘postoperative complication/’ was added to the search strategy and the phrase ‘procedure‐related’ was added in the title and abstract. After adding the Emtree term ‘postoperative complication/’ and the search term ‘procedure‐related.ti,ab.’ to the search strategy created from the development set of records the revised strategy then retrieved 103/116 records (89%) of the records in this evaluation set. Of the 13 records not retrieved by the search strategy, seven had terms related to specific adverse effects (such as ‘postoperative pain’, ‘surgical mortality’, ‘wound infection’). A search strategy which incorporates both generic and specific adverse effects terms could therefore potentially achieve 95% (110/116) recall in the evaluation set of records.

#### Validation of the Embase search strategy

This revised search strategy (Box 3) was then tested on the validation set of records and retrieved 107/116 (92%) of the records. We conducted post‐hoc analysis to identify factors that may have affected the recall. When we explored the records that had not been retrieved from the validation set, there were no additional terms in the records not retrieved that were indicative of generic adverse effects. However, adverse effects specific to the individual paper (such as ‘wound infection’, ‘surgical site infection’) were present in three of the nine records not retrieved by this search strategy. A search strategy which incorporates both generic and specific adverse effects terms could therefore potentially achieve 95% (110/116) recall in the validation set of records.

Box 3 **Final Embase search strategy**

1 complication*.ti,ab.

2 co.fs. [Complication]

3 safe*.ti,ab.

4 ae.fs. [adverse drug reaction]

5 post‐operative morbidity.ti,ab.

6 surgical risk/

7 complication/

8 post‐operative complication/

9 procedure related.ti,ab.

10 or/1–9

The terms which gave the highest precision in Embase were estimated to be the Emtree term ‘complication/’, followed by ‘postoperative morbidity’ or ‘procedure related’ in the title and abstract, then Emtree terms ‘surgical risk/’ and ‘postoperative complication/’.

In summary therefore, the proposed medline search filter in Box 1 retrieved 86%, 94% and 87% of the relevant records in the developmental, evaluation and validation sets of records. The proposed Embase filter in Box 3 retrieved 88%, 89% and 92% of the relevant records in the developmental, evaluation and validation sets of records (Table 3).

**Table 3:** Performance of the search strategies

	Search terms	Development set of records (%)	Evaluation set of records (%)	Validation set of records (%)
medline	Box 1	86	94	87^a^
	Box 1 with specific adverse effects terms	94	96	93
Embase	Box 3	88	89	92
	Box 3 with specific adverse effects terms	91	95	95

a If the search term ‘procedure‐related’ in the title and abstract had been added to the search strategy this recall would have increased to 88%.

>90% sensitivity is represented by green shading.

<90% sensitivity is represented by red shading.

In each case the addition of specific adverse effects terms could have improved the recall of the searches. In medline with the addition of specific adverse effects terms the recall would have been 94%, 96% and 93% in the developmental, evaluation and validation sets of records, respectively. In Embase with the addition of specific adverse effects terms the recall would have been 91%, 95% and 95% in the developmental, evaluation and validation sets of records, respectively (Table 3).

## Discussion

We have used a cohort of published surgical studies to derive and validate a novel search filter for the adverse effects of surgical interventions. The results here give a clear indication of the relevant search terms that work in terms of relative recall performance. Use of the filter will also increase the precision of searches for adverse effects. The recall of searches using solely generic adverse effects terms was 87% in medline and 92% in Embase. With the addition of specific adverse effects terms (to the generic adverse effects terms) the recall could be raised to 93% in medline and 95% in Embase.

Search filters vary in the level of sensitivity that can be achieved. Whilst we strive for 100%, generally any sensitivity above 90% is deemed acceptable (Beynon et al., 2013). This is because some relevant records do not have any terms in the title, abstract or indexing to indicate they met certain criteria and examination of the full text will always be required. In addition there is always a trade‐off between sensitivity and precision. The results here are comparable to search filters for drug intervention adverse effects whereby sensitivity approaching 90% in both medline and Embase was achieved without specific named adverse effects and 93% in medline and 96% in Embase when specific adverse effects terms were added (Golder & Loke, 2012b). However, it should be noted that the results here when searching medline with only generic adverse effects terms in the validation set did not meet the 90% or higher target for sensitivity. Neither did the searches with only generic adverse effects terms in Embase in the developmental or evaluation set meet the 90% or higher target for sensitivity.

Whilst we do not recommend these surgical adverse effects filters be used blindly, we do anticipate that they will assist searchers when devising search strategies to identify relevant studies for a systematic review of surgical interventions. In addition we demonstrate the value of the addition of specific adverse effects terms where possible.

We were also able to compile a list of some of these specific terms commonly used in the databases and we recommend that searchers look to augment the search filter with these specific named adverse effects where appropriate. In particular there are some indexing terms that are specific to surgical adverse effects or suggestive of adverse effects data being available such as the MeSH terms: ‘postoperative hemorrhage/’, ‘postoperative nausea and vomiting/’, ‘pain/’, ‘postoperative, shock/’, ‘surgical/’, ‘surgical wound dehiscence/’, ‘surgical wound infection/’, ‘surgical wound/’*, exp postoperative complications/*in medline. For Embase, the specific terms Emtree terms were: ‘postoperative cognitive dysfunction/’, ‘postoperative delirium/’, ‘postoperative edema/’, ‘postoperative hemorrhage/’, ‘postopertive ileus/’, ‘postoperative ileus/’, ‘postoperative infection/’, ‘postoperative inflammation/’, ‘postoperative nausea/’, ‘postoperative nausea and vomiting/, ‘postoperative pain/’, ‘postoperative thrombosis/’, ‘postoperative vomiting/’, ‘surgical injury/’, ‘surgical infection/’, ‘surgical mortality/’, ‘surgical stress/’, ‘surgical wound/’.

### Limitations

There are two major limitations of the methodology used in this study. The first is the limited number of papers in the developmental, evaluation and validation sets. The second is the lack of a true measurement of precision. We would need a large set of non‐relevant records in order to identify not just the most frequently occurring relevant terms but also the most discriminating terms and to measure precision. The current study simply indicates the relative rank precision of terms in relation to one another.

The next steps in this area need to be the testing and validation on systematic review case studies (in which precision can be measured) and further research with larger sample sizes of relevant papers.

## Conclusions

This is the first proposed search filter for surgical adverse effects. The use of search filters for the adverse effects of surgical interventions is both feasible and advisable in instances where unmanageable numbers of records would otherwise be retrieved. Sensitivity with the proposed generic adverse effects search filters achieved 87% in medline and 92% in Embase and 93% in medline and 95% in Embase when specific adverse effects search terms are added.

Further research on larger datasets is required in order to measure the precision of searching for adverse effects of surgical interventions and to test the suggested search filters with more rigour.

## Conflict of interest

We declare that we have no conflicts of interest.

## Funding

This report is independent research arising from a Postdoctoral Research Fellowship, Su Golder PDF‐2014‐07‐041 supported by the National Institute for Health Research. The views expressed in this publication are those of the authors and not necessarily those of the NHS, the National Institute for Health Research or the Department of Health.
